# Enhancement of Mechanical and Thermal Properties of Oil Palm Empty Fruit Bunch Fiber Poly(butylene adipate-*co*-terephtalate) Biocomposites by Matrix Esterification Using Succinic Anhydride

**DOI:** 10.3390/molecules17021969

**Published:** 2012-02-16

**Authors:** Samira Siyamak, Nor Azowa Ibrahim, Sanaz Abdolmohammadi, Wan Md Zin Bin Wan Yunus, Mohamad Zaki AB Rahman

**Affiliations:** 1 Department of Chemistry, Faculty of Science, University Putra Malaysia, 43400 UPM Serdang, Selangor, Malaysia; Email: s.abdolmohammadi@yahoo.com (S.A.); mzaki53@gmail.com (M.Z.A.B.R.); 2 Department of Chemistry, Center for Defence Foundation Studies, National Defence University of Malaysia, 57000, Malaysia; Email: wanmzin@upnm.edu.my

**Keywords:** oil palm EFB fiber, poly(butylene adipate-co-terephtalate), biocomposite, esterification, peroxide, thermal and mechanical properties, succinic anhydride

## Abstract

In this work, the oil palm empty fruit bunch (EFB) fiber was used as a source of lignocellulosic filler to fabricate a novel type of cost effective biodegradable composite, based on the aliphatic aromatic co-polyester poly(butylene adipate-co-terephtalate) PBAT (Ecoflex^TM^), as a fully biodegradable thermoplastic polymer matrix. The aim of this research was to improve the new biocomposites’ performance by chemical modification using succinic anhydride (SAH) as a coupling agent in the presence and absence of dicumyl peroxide (DCP) and benzoyl peroxide (BPO) as initiators. For the composite preparation, several blends were prepared with varying ratios of filler and matrix using the melt blending technique. The composites were prepared at various fiber contents of 10, 20, 30, 40 and 50 (wt %) and characterized. The effects of fiber loading and coupling agent loading on the thermal properties of biodegradable polymer composites were evaluated using thermal gravimetric analysis (TGA). Scanning Electron Microscopy (SEM) was used for morphological studies. The chemical structure of the new biocomposites was also analyzed using the Fourier Transform Infrared (FTIR) spectroscopy technique. The PBAT biocomposite reinforced with 40 (wt %) of EFB fiber showed the best mechanical properties compared to the other PBAT/EFB fiber biocomposites. Biocomposite treatment with 4 (wt %) succinic anhydride (SAH) and 1 (wt %) dicumyl peroxide (DCP) improved both tensile and flexural strength as well as tensile and flexural modulus. The FTIR analyses proved the mechanical test results by presenting the evidence of successful esterification using SAH/DCP in the biocomposites’ spectra. The SEM micrograph of the tensile fractured surfaces showed the improvement of fiber-matrix adhesion after using SAH. The TGA results showed that chemical modification using SAH/DCP improved the thermal stability of the PBAT/EFB biocomposite.

## 1. Introduction

Responding to globally growing socio-ecological concerns and aiming to diminish the sum of landfill waste and carbon footprints, the thermoplastic industry has focused on developing new environmentally friendly products through mixing biodegradable polymers with natural fibers [1]. Different markets, including packaging, disposable non-woven hygiene products, consumer goods and agricultural tools exist for biodegradable polymers (and hence biocomposites). Eco-aware consumers would see differentiating value in a labeled “green” product. However, biodegradable polymers’ high cost compared to that of cheap petroleum-based non-biodegradable polymers, as well as their limited thermo-mechanical properties, are barriers that need to be addressed for biodegradable polymers in order to be commercially applied in the mass production of commodities. Our goal is to create a biocomposite, which is both financially and thermo-mechanically on-par with conventional thermoplastic composites.

Melt blending biodegradable polymers with biofillers could effectively contribute to the improvement of thermo-mechanical properties as well as to the cost reduction, while at the same time keeping the overall biodegradability features. In order to obtain satisfactory interfacial adhesion, minimum size dispersed phases, and enhanced stress transfer among the components, a proper compatibilizer must be used to control the interfacial energy during the melt blending procedure [1].

Among commercially available biodegradable polymers, biopolyesters have shown similar properties to conventional non-biodegradable polymers. Some aliphatic-aromatic polyesters are considered the most important class of synthetic biodegradable polymers available in a variety of types. For instance, poly (3-hydroxybutyrate-co-3-hydroxyvalerate) Biopol^®^, poly (butylene succinate-co-adipate) Bionolle^®^ from Showa Highpolymers, or copolyesters based on 1,4-butanediol adipic acid and terephatalic acid (Eastar-Bio^®^ and Ecoflex^®^). The main advantage of these polymers is the wide diversity of mechanical and physical properties that are comparable to polymers such as low and high-density polyethylene (LDPE, HDPE) and polypropylene (PP). Poly(butylene adipate-co-terephtalate) (PBAT) is a biodegradable copolyester supplied by BASF and Eastman Chemical Company under the trade name of Ecoflex^®^. It is an aliphatic-aromatic copolyester of butylene glycol and adipic and terephthalic acids which, its high flexible nature and fast degradability when it is deposed in soil with the aid of naturally occurring enzymes, make it extremely suitable for various industrial applications such as food packaging and agricultural film applications [2].

The reinforcement effect of natural fibers on thermoplastic resin matrix has been extensively investigated over the past decades. Superior mechanical properties, biodegradability, cost efficiency, low density, excellent sound absorption, higher shatter resistance, and efficient energy consumption are some of the benefits of natural fibers over glass-carbon and glass fiber reinforced composites. Consequently, researchers have broadly mixed or replaced such natural fibers as kenaf, oil palm, sisal and so on with wooden derivatives and organic resin reinforced matrix. Oil palm fruit bunch (OPFB) fiber that is extracted from empty fruit bunch (EFB) is abundantly found in Malaysia, where it is traditionally used for making ropes, mats, carpets, cushion filler, and others [3].

Hence, there is great potential in turning this fiber into value added products such as using it as reinforcement or filler in bioplastics to manufacture green composites [4]. Chemical analysis of oil palm EFB shows this lignocellulosic fiber consists of 65% cellulose and 19% lignin. Oil palm fiber has relatively high strength and stiffness, low density and causes no skin irritations on contact [5].

Many investigations have been made on the potential of natural fibers as reinforcement for biocomposites in which, biodegradable fibers are used as a reinforcement for biodegradable thermoplastics by means of lignocellulosic fibers that significantly improve stiffness and strength while drastically reducing the composite toughness [5]. 

However, the primary drawback of using natural fibers as reinforcements in biocomposites is the incompatibility between the polar-hydrophilic biofibers and non polar-hydrophobic bio-thermoplastic matrices and hence poor interfacial interaction between them. Bledzki and Gassan have stated that the quality of the fiber-matrix interface is significant for the application of natural fibers as reinforcements for bioplastics. It requires the use of compatibilizers or coupling agents in order to improve the adhesion between fiber and matrix [3].

Biopolymer surfaces, acting as boundaries lying between the external environment and the bulk polymer, directly influence a biopolymer’s performance. The untreated non-polar polymer surfaces often associated with the hydrophobic nature of most biopolymers, results in limitations regarding the coloring, lamination, packaging, coating, adhesion and colloid stabilization. To solve these problems, an enormous amount of basic and applied research has been devoted to the surface modification of biopolymer materials [6].

Until recently, a number of different innovative techniques, including chemical methods and physical processes, have been studied and applied in various industrial application fields to optimize the interfacial interaction between lignocellulosic fillers and bio-thermoplastic polymers [6]. Physical modifications intend to change the structure and surface of the fiber and thereby influence the mechanical bonding with resins, while chemical treatment involves modification of hydroxyl groups and introduces new ions that can effectively interlock the matrix [7]. Chemical modification methods such as modification of fiber by free radical reactions, addition of a third component with the ability to interact simultaneously at the composite’s interface with filler and matrix (compatibilizer), and the use of surface-modified thermoplastics containing a compound capable of interacting with filler [5] are of different efficiency for the adhesion between matrix and filler.

The use of different kinds of surface treatment methods leads to changes in the surface structure of the fibers as well as matrices. The type and amount of surface modifier and processing conditions must be optimized both from a technical and economical point of view. There are several chemical methods to introduce a third material (coupling agent), that has properties intermediate between hydrophilic filler and hydrophobic matrix to achieve a good compatibility in composites, such as graft co-polymerization and coating treatments [8]. 

Generally, coupling agents facilitate the optimum stress transfer at the interface between fiber and matrix [9]. Coupling agents are categorized into three major groups, namely; organics (including anhydrides, amides, imides, organic acids, monomers, polymers, copolymers and many others), inorganics and organic-inorganics, with just a few inorganics such as silicates applied in biocomposites. Silanes are widely used as organic-inorganic coupling agents in natural fiber reinforced biocomposites. Anhydrides such as maleic anhydride (MAH), acetic anhydride (AAH) and succinic anhydride (SAH) are popular coupling agents with two functional groups [carboxylate groups (–COO–)], capable of linking biofibers through hydrogen bonding or esterification. Multifunctional groups such as [–(CO)_2_O–] of succinic anhydride, existing in organic coupling agents, tend to interact with the polar [mainly hydroxyl groups (–OH)] of cellulose and lignin, forming covalent or hydrogen bonding. 

Organic peroxides such as benzoyl peroxide (BPO) and dicumyl peroxide (DCP), can potentially initiate free radical reactions between cellulosic or lignocellulosic fibers and polyethylene matrix systems. Organic coupling agents also can modify the polymer matrix by graft copolymerization, thus resulting in strong adhesion, even crosslinking, at the interface [3,10,11].

A thorough literature study revealed that no work has been so far done on the chemical modification of poly(butylene adipate-co-terephtalate) composite incorporating oil palm empty fruit bunch fiber, using SAH as coupling agent to produce a novel biodegradable composite with acceptable thermal and mechanical properties. 

Hence, this research aimed to develop EFB particle-reinforced PBAT biocomposite using the melt blending technique. The PBAT/EFB fiber biocomposites were characterized and analyzed for their mechanical, thermal, and morphological properties. In the first stage, we focused on examining the viability of using EFB fiber as filler to produce biocomposites. Subsequently, the influence of different amount of SAH in presence and absence of different types and amounts of peroxides as initiators on the filler-matrix compatibility were investigated.

## 2. Results and Discussion

### 2.1. FTIR Spectroscopy

**Figure 1:**
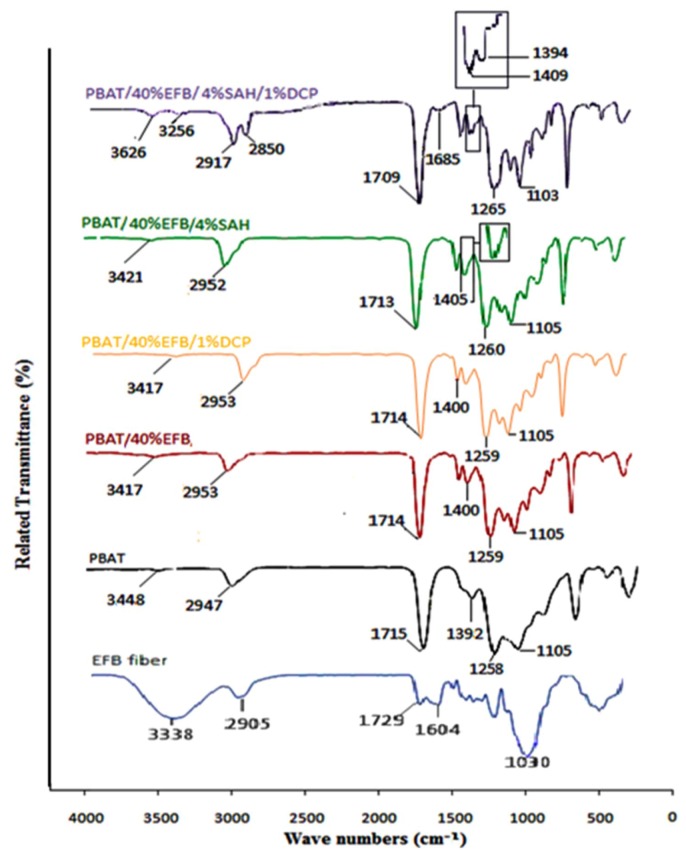
FTIR spectra of the PBAT and its composites filled by EFB fiber before and after chemical modification using SAH and/or DCP initiator.

Since the interactions of polymer composites can be identified by FTIR spectroscopy, FTIR analysis was used to investigate the grafting reaction of SAH onto PBAT and to verify the formation of ester bonds at the interface. Figure 1 shows the FTIR spectra of PBAT/EFB composites at room temperature in several specific stretching regions. PBAT had a strong carbonyl stretching absorption at 1,715 cm^−1^. With the addition of 40 (wt %) of EFB fiber and also with addition of 4 (wt %) of SAH, the C=O peak of PBAT at 1,715 cm^−1^ shifted to a lower wave number, respectively. The C=O peak of PBAT even shifted to a lowest wave number by 7 cm^−1^ to 1,709 cm^−1^ after addition of 4 (wt %) of SAH and 1 (wt %) of DCP. The interaction formation would change the stretching vibration frequency of the carbonyl bound and give rise to a shift [12]. It meant that carbonyl groups of PBAT could form some chemical interaction with hydroxyl groups of EFB in presence of SAH/DCP.

According to the IR spectra of PBAT/EFB composites, the interaction between EFB filler and PBAT matrix without chemical treatments could be a physical interaction as there is no new band or any significant shift compared to the PBAT spectrum. In addition, after adding SAH the improved mechanical and physical properties of PBAT/EFB composites could be due to the chain entanglement of fiber particles-polymer matrix, which improved fiber-matrix interfacial strength. Free chain ends of two polymers can diffuse at the interface providing chain entanglement and mechanical interlocking [13], rising interfacial strength. This effect is employed in some coupling agents used on fibers in thermoplastic matrices [8]. It must be noticed that the PBAT/EFB/DCP composite spectrum did not show any changes compared to the PBAT/EFB composite’s spectrum which indicates that addition of DCP to the PBAT/EFB composite did not promote any chemical interaction in the composite. 

Based on composites spectra presented in Figure 1 and according to [12,13,14], the carbon-oxygen (–O–C–O–) stretching modes gave rise to intense and complex multiple peaks in the region between 1,000 cm^−1^ and 1,300 cm^−1^. The carbon-oxygen (–O–C–O–) stretching vibration appeared at 1,258 cm^−1^ in the virgin PBAT spectrum, which deviates with addition of SAH/DCP by 7 cm^−1^ and appears at 1,265 cm^−1^ in the PBAT/EFB/SAH/DCP. 

It meant that SAH in the presence of DCP enhanced the interaction between PBAT and EFB fiber, which was related to carbon-oxygen groups such as, C=O, –O–C–O– and C–O [12]. The band of frequency between 1300–1500 cm^−1^ (1375 cm^−1^), usually used to characterize a methyl group [15], also appeared in the SAH/DCP modified PBAT/EFB fiber composite spectrum, which could be an evidence of esterification reaction at the interface and presence of new covalent bonds. Compared with the PBAT spectrum, another new band appeared at 1,685 cm^−1^, which confirmed the presence of carbonyl groups in the PBAT/EFB fiber composite after addition of SAH/DCP. In the PBAT-g-SAH molecules, two different carbonyl functional groups separated by two carbon atoms that do not lie in the same plane, could be assigned to individual keto and acid groups. 

According to Li [16], under this condition conjugation with an aromatic group leads to a lower frequency, thus the keto group absorbs at 1,685 cm^−1^, while the free acid exhibited bands at 1,710 cm^−1^ which are attributed to the absorbance of isolated and hydrogen-bonded carbonyl groups. Peaks near 2,900 cm^−1^ could be assigned to the carbon-hydrogen (C–H) stretching [15].

Therefore, the presence of two new peaks at 2,917 cm^−1^ and 2,850 cm^−1^ in the spectrum of SAH/DCP-treated composite indicates the presence of the aliphatic chain arising from grafting. Furthermore, the SAH grafted polymer composite in the presence of DCP, exhibited a peak between 3,330 cm^−1^ corresponding to an hydroxyl group (–OH) stretch, which was also observed in EFB fiber, but not in PBAT. This peak can be attributed to primary alcohols [1] and its appearance in the IR spectrum of grafted composite reveals further interaction between fiber and matrix after chemical modification. 

**Scheme 1:**
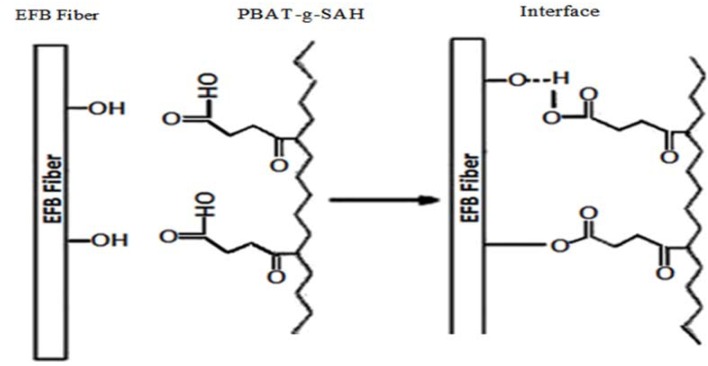
Hypothetical reaction mechanism at the matrix-fiber interface with chemical modification.

Both Pandey and Wu [17,18] deduced that the spectrum band near 3,330 cm^−1^ arises from hydroxyl groups (–OH) in the polymeric structure and since it is present at comparatively lower frequency than the free hydroxyl group (3,400–3,600 cm^−1^), this is due to intermolecular hydrogen bonding by the interaction between the carboxylic acid group (–COOH) of anhydride and the hydroxyl groups (–OH) of fiber. According to the IR spectrum of SAH-modified PBAT/EFB biocomposite in the presence of DCP initiator, compared to the untreated PBAT/EFB biocomposite, we can conclude that there are different chemical primary (covalent bonds) and secondary (hydrogen bonds) interactions between fiber and matrix after chemical treatment. The possible reaction mechanism between SAH, matrix and fiber with chemical modification is presented in Scheme 1, which is supported by the FTIR spectra presented in Figure 1.

### 2.2. Effect of Fiber Loading on Tensile Properties of Biocomposites

**Figure 2:**
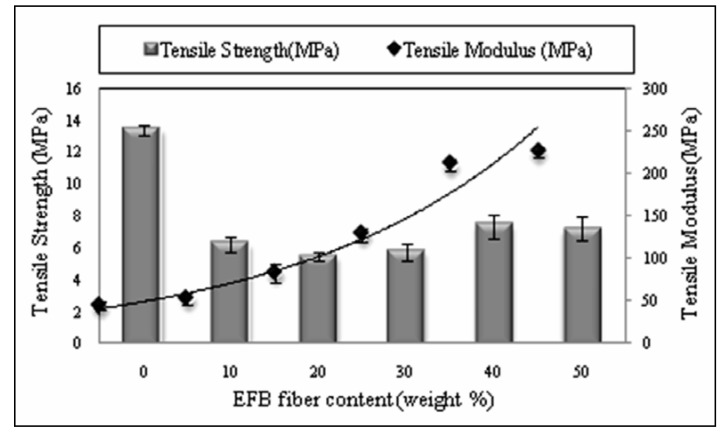
Effect of fiber content on tensile properties of PBAT biocomposites.

PBAT/EFB composites with different fiber content were fabricated in order to determine the best formulation of the biocomposite. Figure 2 depicts the variation of the tensile strength as well as tensile modulus as a function of oil palm EFB fiber loading. Addition of EFB fiber decreased the tensile strength of PBAT, although there is a moderate increase in tensile strength of composite at 40% of fiber loading and beyond this point, tensile strength decreased with the increase of fiber content. The highest tensile strength of 7.5 MPa was observed at 40% of fiber loading. 

The decline in tensile strength with increasing fiber content could be due to the lack of enough PBAT to wet EFB fiber, resulting in an inefficient stress transfer [19]. This also might be due to the incompatibility of hydrophilic lignin in the EFB fiber with the matrix, leading an easy composite failure [20,21]. Generally, the increase in tensile strength (at 40% fiber loading), indicates the ability of EFB fiber to absorb stress transferred from the PBAT matrix. In composites with higher fiber content, there is a greater tendency for filler-to-filler interaction to take place [22], therefore, more voids are formed, initiating the crack formation and propagation in the composite, compared with low fiber loadings. Figure 2 also clearly illustrates that the biocomposites tensile modulus was considerably enhanced by fiber loading at high fiber content, as the increased fiber population leads to agglomeration, which affects the biocomposites’ stiffness. The overall increase in the modulus demonstrates the ability of the EFB fibers to impart greater stiffness to the composite [23]. Composite at 50 (wt %) of fiber content showed the highest tensile modulus of 225 (MPa).

**Figure 3:**
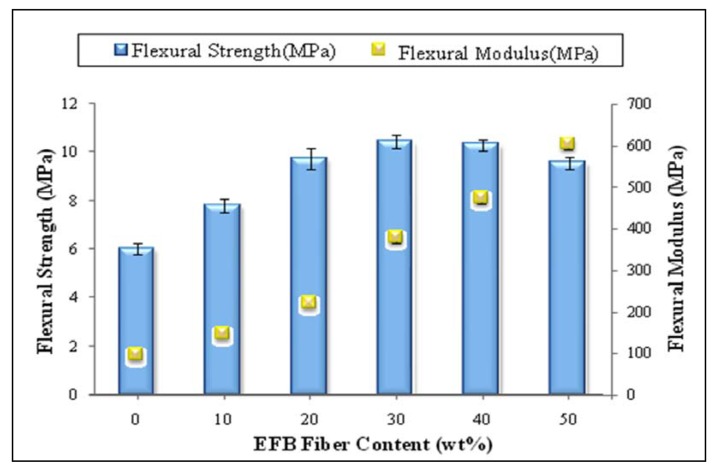
Effect of fiber loading on the flexural properties of PBAT composites.

Figure 3 depicts the effect of fiber loading on flexural strength and flexural modulus of oil palm EFB fiber reinforced PBAT biocomposites. It is clear that both flexural strength and modulus show a continuous increase with the increase of EFB fiber loading, although there is a slight decrement in flexural strength from 40% to 50% fiber loaded biocomposite. 

The increase in flexural strength with fiber loading may be due the ability of the EFB fiber to absorb the stress transferred from the matrix or the improvement in compatibility between the fiber and matrix because of better fiber distribution in the matrix. However, the increased fiber loading from 40% to 50% slightly decreased the flexural strength of composite. In fact, small fiber particles have high tendency of agglomeration due to the surface energy or Van der Waals attraction, where it depends on the surface area. Higher surface area results in higher surface energy [4] and poor fiber/matrix adhesion. Figure 3 shows that 40 (wt %) of EFB fiber loading has significantly increased flexural strength and modulus of PBAT biocomposite by about 87% and 412%, respectively. This indicates that, as the modulus is a measure of flexural stiffness of composite, incorporation of fillers is able to improve the stiffness of composite. Similar results were observed by Sykacek on their study on the mechanical performance of different biopolymers including PBAT biocomposites reinforced with biofillers [24]. 

### 2.3. Effects of Chemical Modification on Tensile Properties of PBAT/EFB Biocomposites

**Figure 4:**
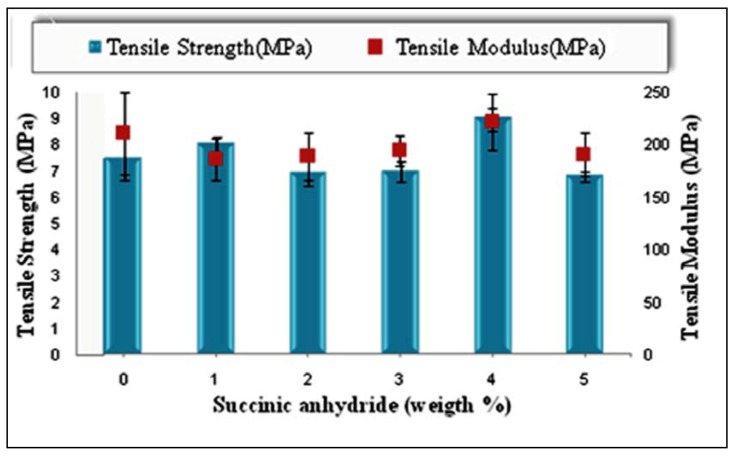
Effect of SAH content as a coupling agent on the tensile properties of PBAT composite reinforced with 40 (wt %) of EFB.

Figure 4 illustrates the effect of chemical modification using SAH on tensile strength and modulus of PBAT biocomposites at 40% EFB fiber loading. It was observed that 4 (wt %) of SAH based on total fiber weight in composite, gives the highest tensile strength at 8.9 (MPa) as well as tensile modulus at 220 (MPa). This result indicates that SAH shows its best result in coupling efficiency at 4 (wt %) concentration, with an increase about 20.32% and 5.24% in tensile strength and modulus respectively, compared to the untreated composite by improve the interfacial adhesion. 

This may be due to the sufficient amounts of SAH and its appropriate M_w_ allows better diffusion into the polymer matrix, which indicates easier entanglement with the matrix polymer [25]. However, there is a decrease in tensile strength with a further increase in the concentration of SAH in biocomposites. This might be due to the fact the excess amount of coupling agent could not react with fiber or matrix and affected the interface interaction and resulted in tensile properties decline [10]. This also could be due to the coupling agent lower molecular weight compared to the matrix, which seems to be responsible for plasticizing effect [26]. Figure 4 also shows that the tensile modulus was marginally improved by increasing the SAH content as a coupling agent up to 4 (wt %) of SAH from 210 (MPa) for untreated composite to 221 (MPa), and beyond this point the composite with higher amounts of SAH shows a decrease in tensile modulus. The increase in tensile modulus of the composites using coupling agent is showing the sufficient lubricant effect of coupling agent to help the matrix to show better fiber packing effect which leads to less voids and consequently to form a strong composite with high rigidity and desired strength properties [27]. At high SAH content, there is a decrease in tensile modulus trend. The reason that higher coupling agent concentrations result in lower mechanical properties of the composite possibly lies in the formation of different by-products, increase in concentration of unreacted or ungrafted coupling agents, and interference with coupling reaction. Consequently, an excess of a coupling agent may act as an inhibitor rather than a promoter of adhesion [10]. 

#### 2.3.1. Effect of Type and Amount of Initiator on Tensile Properties of PBAT/EFB Biocomposites

**Figure 5:**
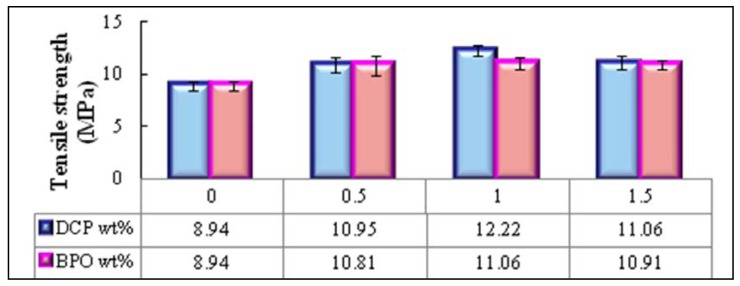
Effects of initiator content and type on the tensile strength of PBAT/EFB biocomposite in presence of 4 (wt %) of SAH.

**Figure 6:**
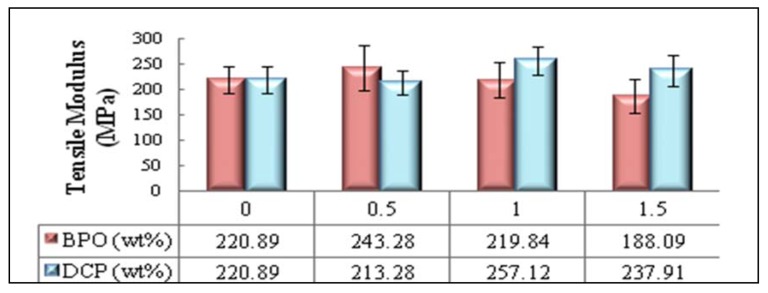
Effects of initiator type and content on the tensile modulus of PBAT/EFB biocomposite at 4% SAH loading.

Further experiments were carried out to study the effect of type and amount of organic peroxide as initiator on the mechanical properties of PBAT/EFB fiber biocomposite treated by 4 (wt %) of SAH. DCP (dicumyl peroxide) and BPO (benzoyl peroxide) are well known initiators have been widely used in free-radical melt grafting of anhydrides onto polyesters. Figure 5 and Figure 6 illustrate the effect of type and contents of various organic peroxides as initiators on tensile strength and modulus of EFB fiber reinforced PBAT biocomposites, respectively.

From Figure 5, it is obvious that 1 (wt %) DCP showed the best result in tensile strength of new biocomposite. There was a large increase in the tensile strength of PBAT/EFB fiber biocomposite (closely 36.6%) showing that SAH in collaboration of DCP has considerably improved the matrix interfacial adhesion with EFB during the biocomposite preparation. Besides, the 1 (wt %) of BPO initiator improved tensile strength of new biocomposite about 23% compared to the untreated sample, from 8.9 (MPa) to about 11.1 (MPa), which is also a notable improvement. Further addition of BPO did not improve the tensile strength significantly. These results showed a further enhanced compatibility of EFB sites with the PBAT in presence of DCP initiator, which could be due to the better solubility of DCP than BPO in the PBAT matrix [27]. However, with a further increase in the concentration of DCP initiator from 1 (wt %) to 1.5 (wt %) a decrease was observed in the tensile strength of biocomposite. This might be due to the crosslinking/chain branching, which is a competitive common side-reaction in high concentration of organic peroxide, specifically chain branching reaction may be occur when the grafting is promoted using a peroxide, in the free-radical melt grafting of anhydrides onto polyethylene [27]. 

Figure 6 depicts the tensile modulus behavior of new biocomposites initiated with various amount of BPO and DCP initiators. As stated earlier, better adhesion between fiber and matrix can have additional effects on the tensile modulus since interface has a great impact on deformation capacity of composites. There is a moderate increase (about 16%) in the composite treated with 4 (wt %) of SAH and BPO, where BPO concentration is 0.5 (wt %) of fiber weight. The best improvement (about 22.4%) in tensile modulus of new biocomposites achieved where the succinic anhydride grafting process was induced by 1 (wt %) of DCP.

This could be explained by the DCP-promoted grafting of the PBAT/SAH onto the EFB fiber surface that would have destroyed the aggregates from filament particles, and thus improved the dispersion of EFB fiber particles surrounded by PBAT chains. This may result in an increase of surface specificity for the fiber particles, forming a composite with a higher Young’s modulus [28]. Similar results reported by Raquez [28] in their interesting study on poly(butylene adipate-co-terephthalate)/talc biocomposite. In addition, the plasticizing effect of SAH while it is grafted onto PBAT in the presence of high amount of initiator may reduce the composite stiffness and therefore have decreased the composites’ modulus [29]. 

### 2.4. Effect of Chemical Modification Using SAH and Initiator on Flexural Properties of PBAT/EFB Biocomposites

**Figure 7:**
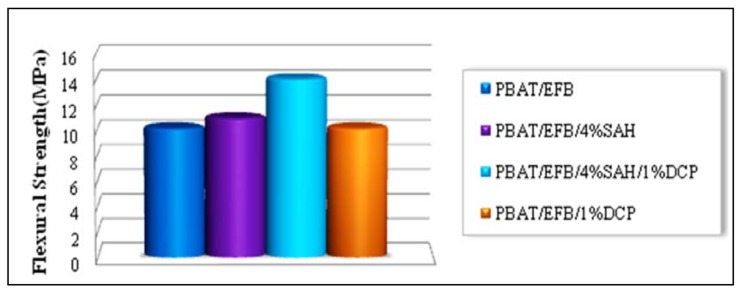
Effect of SAH and DCP initiator on the flexural strength of PBAT/EFB biocomposites.

The effect of chemical treatments using SAH in presence and absence of DCP initiator on the flexural strength of new biocomposites compared to those of the untreated PBAT/EFB composites are shown in Figure 7.

The PBAT composite filled by 40 (wt %) of untreated EFB fiber, based on total weight of composite was assumed as the control sample. The composite prepared at 4 (wt %) of SAH concentration showed a marginal improvement in flexural strength with an increase of only 8%, whereas the composite prepared at 4 (wt %) of SAH in presence of 1 (wt %) of DCP and under the same composite processing conditions showed a considerable enhancement in the flexural strength (about 38%) compared the control sample. This phenomenon is probably due to the enhanced interfacial adhesion between the EFB fibers and the PBAT matrix intensified by SAH when the free radical melt grafting reaction has been initiated by DCP as a free-radical generator. It is clear that the addition of DCP initiator in absence of SAH did not improve the mechanical properties of PBAT/EFB biodegradable composite. 

**Figure 8:**
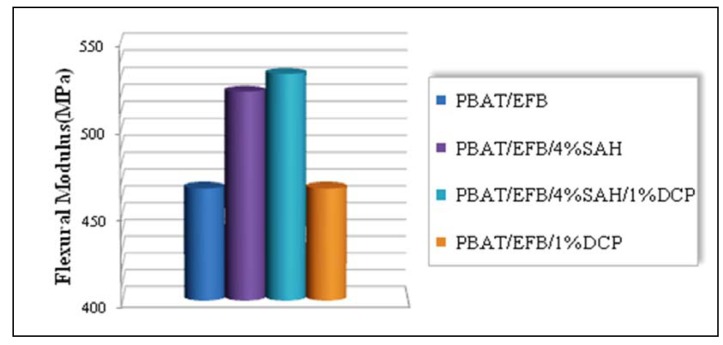
Effect of SAH and DCP initiator on flexural modulus of PBAT/EFB biocomposites.

The flexural modulus of new biocomposites is depicted in Figure 8. It is obvious that excluding the PBAT/EFB composite treated with 1 (wt %) of DCP, all chemical modifications to form new biocomposites have increased the flexural modulus of new biocomposites compared to the control sample. 

The flexural modulus of the composites treated with succinic anhydride coupling agent in presence and absence of DCP initiator, showed an increase about 14% and 12%, respectively, compared to the control sample. Generally, the flexural behavior of modified PBAT/EFB biocomposite indicate that succinic anhydride, as a coupling agent in presence of DCP initiator is able to enhance the stiffness and interfacial bonding strength of the new biocomposite. Based on the achieved results we can conclude that direct coupling of SAH (free radical melt grafting) onto the polymer matrix backbone imparted the greater stiffness and stronger interfacial bonding to the biocomposite. The mechanical strength improvement is primarily attributed to the efficient homogeneity of thermoplastic polymer matrix, reinforcing effect and good dispersion of fiber in the matrix that inhibited the agglomeration and allowed a uniform stress distribution [30] from the PBAT matrix to the EFB filler. 

Consequently, chemical modification of biocomposite, using succinic anhydride as coupling agent and organic peroxides to initiate the graft mechanism in modification procedure, enhanced the fiber matrix interfacial interaction by improving their compatibility. It has also shown some positive effects on the tensile properties of the new biocomposite compared to the untreated PBAT/EFB biocomposite.

### 2.5. Effect of Fiber Loading and Chemical Modification on Thermal Properties of PBAT/EFB Biocomposites

#### 2.5.1. Effect of Fiber Loading on the Thermal Properties of PBAT/EFB Fiber Composites

**Figure 9:**
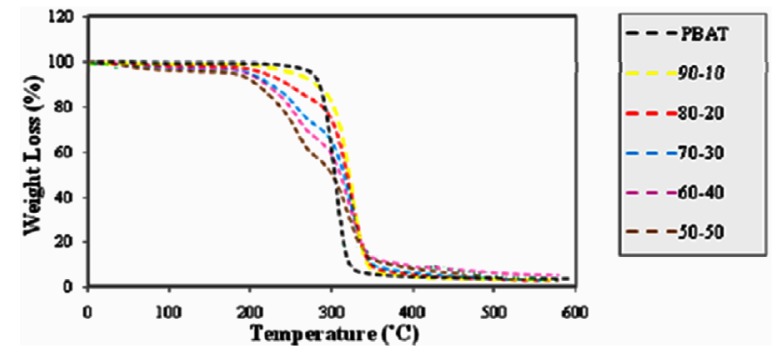
Effect of fiber content on thermal degradation of PBAT/EFB biocomposites (TG thermograms).

**Figure 10:**
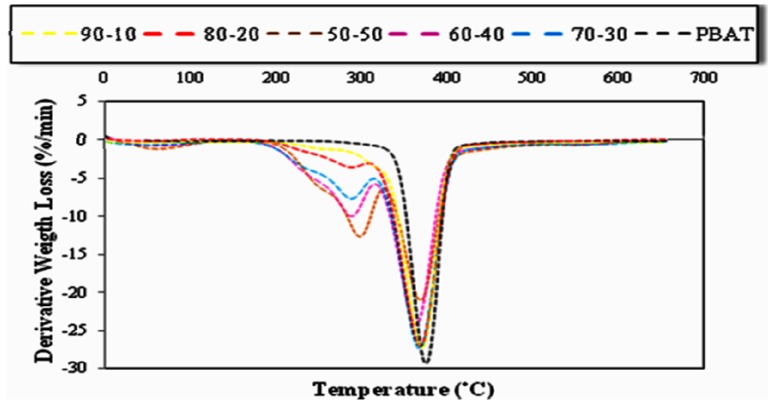
DTG thermograms of PBAT and PBAT/EFB composites in various fiber contents.

The DTG and TG data and thermograms of EFB fiber reinforced PBAT biocomposites with different fiber contents are presented in Table 1 and Table 2 as well as Figure 9 and Figure 10, respectively**. **Thermal stability of composites declined as the fiber content in composites increased. It is obvious that the devolatilization of moisture of EFB fibers occurred in temperature range of 70–100 °C for even after the EFB fiber incorporated into the PBAT matrix. Such moisture content might play a significant role in degradation processes due to the fact that OH group in water is more reactive than the OH groups available in the EFB fiber. The thermal degradation of EFB fiber was due to the decomposition of cellulose, lignin and hemicellulose to give off volatiles [31]. PBAT starts to decompose at about 310 °C. The mass loss of PBAT in a one-step degradation procedure starts at about 310 °C and continues very slowly until 350 °C; at temperatures beyond 350 °C, this progression occurs quickly. The amount of PBAT residue is about 4.4% because of its further breakdown into gaseous products at high temperature (Table 1).

Alternatively, the thermal degradation of the PBAT/EFB composites with various fiber contents takes place in a two-step degradation process. Besides, mainly due to the decomposition of EFB fiber, the PBAT/EFB biocomposites exhibited initial mass loss from approximately 207 °C to 340 °C. Then, the second thermal degradation step, which is mainly related to PBAT degradation, overlapped with cellulose and lignin content in EFB fiber is observed. This two-step degradation process demonstrates that the thermal degradation temperature of the PBAT is higher than of the EFB fiber. 

**Table 1:** Summary of DTG_max_ degradation temperature of PBAT and PBAT/EFB biocomposites with various fiber contents.

Specimens	First peak (°C)	Second peak (°C)	Third peak (°C)
PBAT	□	□	379.6
PBAT/EFB10%	□	□	386.1
PBAT/EFB20%	‒	305.5	383.3
PBAT/EFB30%	88.0	307.5	382.2
PBAT/EFB40%	88.8	307.0	380.9
PBAT/EFB50%	79.5	307.2	379.4

Furthermore, Table 2 clearly shows that, the formation of oil palm EFB biocomposites using hydrophobic PBAT matrix reduced water content of biocomposites. PBAT surrounds EFB fiber and its presence, reduces fiber hydrophilicity and decreases the water content in biocomposite by preventing water from reaching the EFB fiber.

**Table 2:** Summary of TGA data for PBAT and PBAT/EFB biocomposites with various fiber contents.

Sample	Water content (%)	Initial degradation temperature (°C) *	T_10%_	T_50%_	T_80%_	Final degradation temperature (°C)	Ash content (%) **
PBAT	□	311.5	352.8	381.3	398.4	413.3	4.4
PBAT/EFB10%	□	280.4	339.7	382.8	399.2	431.7	6.7
PBAT/EFB20%	1.3	248.8	302.3	378.0	398.6	432.5	9.1
PBAT/EFB30%	2	210.2	276.7	373.6	401.5	440.2	11.2
PBAT/EFB40%	3.9	153.3	275.2	371.1	400.2	443.6	12.2
PBAT/EFB50%	3.4	148.2	233.3	360.2	398.8	447.7	17.7

Note:* The initial degradation temperature considered as the temperature when the sample loses 3% of its weight; ** The ash content is the weight percentage at 550 °C TGA data for PBAT/EFB biocomposites. No “**” mentioned in the table?

From the data presented in Table 2 we can conclude that the intermediate values of degradation temperature suggest again some kind of interaction between the PBAT matrix and fiber. Based on the weight percentage (wt %) of EFB fiber content, the thermal stability of PBAT/EFB fiber composites follows the sequence of 10% > 20% > 30% > 40% > 50% [2].

#### 2.5.2. Effect of Chemical Modification on the Thermal Properties of PBAT/EFB Fiber Composites

Thermogravimetric analysis (TGA) was performed to study the effect of chemical modification using SAH in the presence and absence of DCP initiator on the thermal properties of PBAT/EFB biocomposites. The thermogravimetric (TG) details of the experimental data for PBAT (sample weight loss percentage with respect to the temperature) and its composites filled by 40 (wt %) of EFB fiber before and after chemical modification with SAH in presence and absence of DCP initiator, are presented in Table 3. It is clear that the water content of PBAT/EFB biocomposite decreased after chemical treatments on fiber and/or matrix compared to that of the composite without chemical additive and coupling agent. As stated earlier, forming oil palm EFB biocomposites using hydrophobic (PBAT) matrix reduces the composites’ water content. Moreover, chemical treatments have also reduced the biocomposites’ water content. Chemical treatment turns the fiber surface hydrophobic and fibers’ functional groups are surrounded with hydrophobic PBAT, thus inhibiting water penetration into the composites’ structure. Similar results were reported by Mohanty [32] in their study on the effect of organic modification on the properties of PBAT biodegradable nanocomposites. 

**Table 3:** Summary of TGA data for PBAT/EFB biocomposites with various chemical modifications using SAH/DCP.

Sample	Water content (%)	Initial degradation temperature (°C) *	T_10%_	T_50%_	T_80%_	Final degradation temperature (°C)	Ash content (%) **
Neat PBAT	□	311.5	352.8	381.3	398.4	413.3	4.4
PBAT/40%EFB	3.9	153.3	275.2	371.1	400.2	443.6	12.2
PBAT/40%EFB/4%SAH	1.9	114.7	268.7	373.7	445.9	445.5	13.4
PBAT/40%EFB/4%SAH/1%DCP	1.8	127.4	284.2	378.6	419.8	449.2	14.1

Note:* The initial degradation temperature considered as the temperature when the sample loses 3% of its weight; ** The ash content is the weight percentage at 550 °C.

On the other hand, the thermal degradation data of the PBAT biocomposite reinforced with 40 (wt %) of EFB fiber takes place in a two-step degradation process. The biocomposite exhibits an initial mass loss (about 28.5%) from approximately 200 °C to 330 °C, which is generally due to the decomposition of hemicellulose and cellulose contents of EFB fiber in composite. Following, the second thermal degradation step of PBAT overlapped cellulose and lignin content that is about 51% weight loss, occurred between 330 °C and 430 °C. This two-step degradation process demonstrates that the thermal degradation temperature of the EFB fiber is lower than PBAT’s. 

In addition, to compare the effects of temperature changes on the weight loss rate of different PBAT/EFB composites, derivative thermogravimetry (DTG) were performed and the related data are presented in Table 4. Regarding to Table 4, the slight but significant increase in maximum weight loss temperature of EFB fiber reinforced PBAT composite compare to the neat matrix may counts as the main effect of fiber loading on the thermal properties of new biocomposite. In order to verify the interactions between the degradation mechanisms affecting the ash residues, the weight percentage at 550 °C was measured (Table 3). The char formation, related mainly to the fiber (when pure EFB fiber, producing 29% of char residues, while PBAT produces only 3.8%), follows this order for the materials studied in this work: PBAT/EFB/4%SAH/DCP > PBAT/EFB/4%SAH > PBAT/40%EFB composite. Chemically treated biocomposites presented more ash residues compared to the composite without coupling agent. This behavior possibly occurs because with the increase of the interaction between fiber and matrix in the degradation process, the polymer matrix may contribute along with the fiber in the process of char residue generation [33]. 

**Table 4:** Summary of DTG_max_ degradation temperature of PBAT/EFB biocomposites.

Specimens	First peak (°C)	Second peak (°C)	Third peak (°C)	Fourth peak (°C)
PBAT	□	□	□	379.6
PBAT/40%EFB	88.8	□	307.0	380.9
PBAT/40%EFB/4%SAH	76.7	247.5	309.7	380.4
PBAT40%EFB/4%SAH1%/DCP	76.2	272.0	333.6	390.0

The addition of SAH did not change the degradation temperature of PBAT/EFB fiber composite. From the data presented in Table 3 and Table 4, it is clear that the thermal degradation of untreated composite starts at about 153.3 °C, and finishes at 443.6 °C with a main peak at 380.9 °C. The mass loss before the onset temperature was related to the volatilization of fiber water content. Therefore, the difference of mass loss at onset temperature of the neat PBAT and composites with and without SAH coupling agent was mainly due to the EFB fiber contents and SAH, which had a significant effect on mass loss at onset temperature of treated biocomposite. 

On the other hand, the SAH/DCP-treated PBAT/EFB biocomposite displayed a higher thermal stability as evidenced by the increase in the temperature of maximum degradation rate and the initial mass loss temperature. The decomposition temperature of PBAT was around 380.4 °C, its complete decomposition took place about 400 °C. However, there is an increase in the decomposition temperature of PBAT with addition of fiber (about 380.9 °C) and even more increase (about 395.0 °C) was observed by adding SAH as coupling agent and chemical treatment of the matrix in the new biocomposite in the presence of 1 (wt %) of DCP initiator. As a matter of fact, the presence of dicumyl peroxide initiated the SAH graft reactions onto polymer backbone and increased the possibility of chemical bonding between the functional groups of SAH and PBAT molecules during the SAH grafting reactions via free radical graft copolymerization, and consequently led to higher thermal stability of new biocomposite. This indicates that the thermal stability of the new biocomposite depends on the degree of SAH grafting onto the PBAT matrix. A similar observation was also reported by Rahman, *et al*. [34] in their study on the influence of silane coupling agent on the thermal behavior of HDPE biocomposite reinforced with rice straw in presence and absence of DCP initiator. 

### 2.6. Morphological Study of PBAT/EFB Biocomposite at 40% Fiber Loading

**Figure 11:**
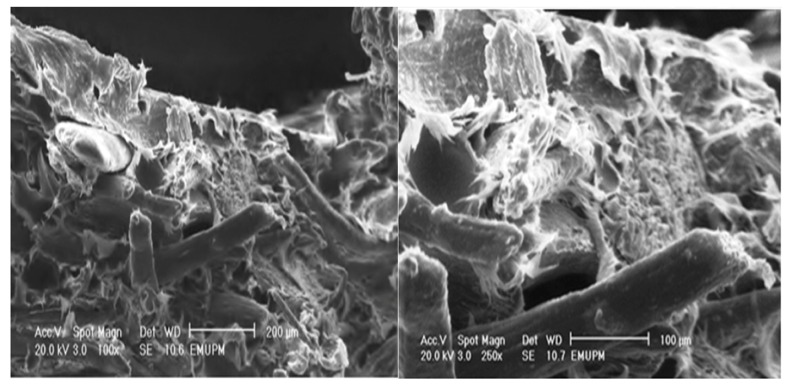
The tensile fracture surface micrograph of PBAT biocomposite reinforced with 40 (wt %) of EFB.

Figure 11 illustrates a scanning electron micrograph of the tensile fracture surface of PBAT/EFB composites at 40 (wt %) of fiber content. At first sight, the dispersion of fibers in the matrix, regardless of the employed surface treatment is clear, proving the efficient mixing of fibers in the matrix via melt blending of fibers and PBAT in Rheomixer. As seen in Figure 9, the EFB fiber composite fracture occurred, predominantly, by transversal fracture in the flat surface of the PBAT matrix. It is observed that the EFB particles in fiber form oriented in random arrangement, and as the adhesion fiber/matrix was poor, the composite shows some gaps and signs of pullout tendency. In addition to fiber pullout, some fiber breakage as the mean of fracture can also be seen in the PBAT/EFB untreated sample.

### 2.7. Morphological Study of PBAT/EFB Biocomposite after Cemical Modification

**Figure 12:**
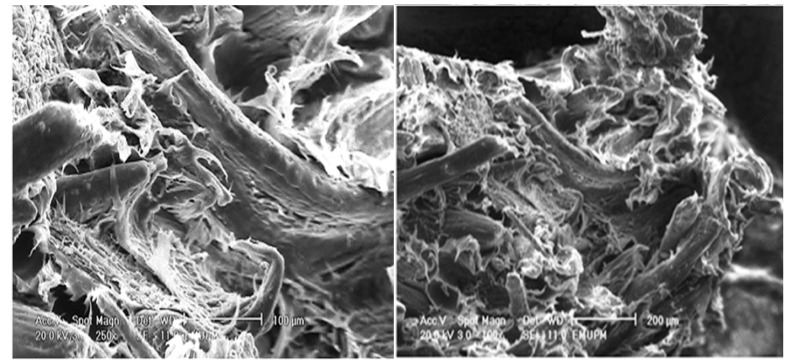
Tensile fracture surface micrograph of PBAT biocomposite reinforced with 40 (wt %) of EFB and 4 (wt %) of SAH.

**Figure 13:**
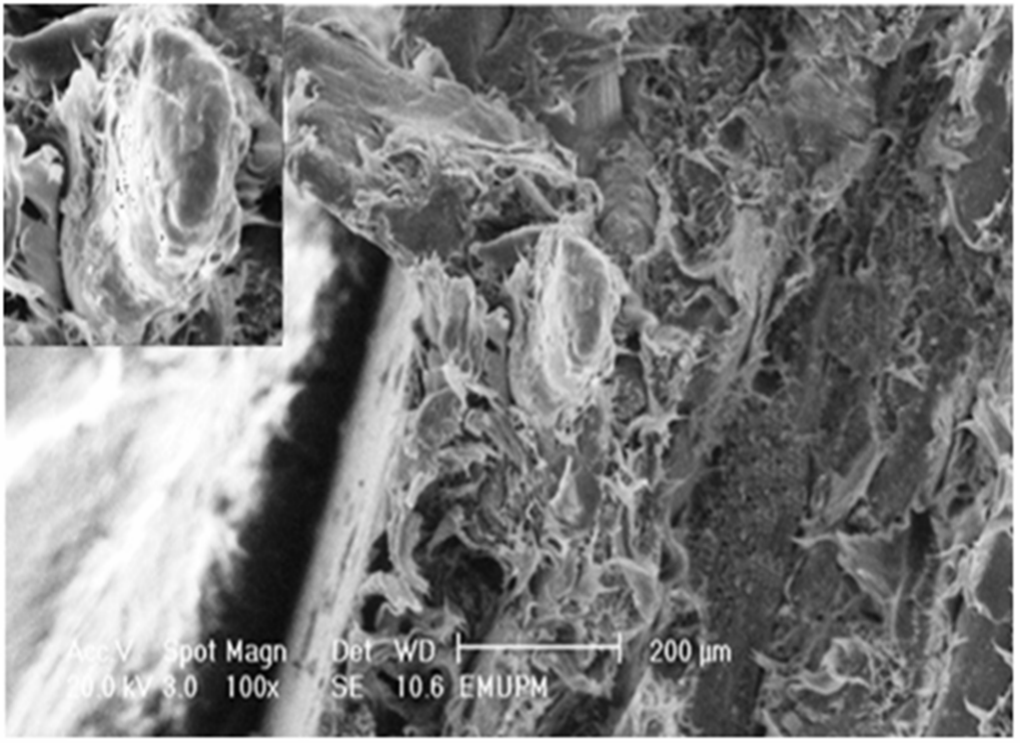
Tensile fracture surface (SEM) micrograph of PBAT biocomposite reinforced with 40 (wt %) of EFB and treated with 4 (wt %) of SAH in presence of 1 (wt %) of DCP.

Figure 12 depicts the effect of chemical treatments using SAH on the fracture surface of PBAT/EFB biocomposites. It was observed that 4 (wt %) of SAH did not cause any significant changes on the fracture surface of 4%SAH/PBAT/EFB composite although there is less fiber pull out and gaps compared to the untreated composite, whereas using 4 (wt %) of SAH in presence of 1 (wt %) of DCP has changed the biocomposite fracture mode significantly. In the light of SEM observations (Figure 13), it is evident that addition of 4%SAH/1%DCP changed the fracture surface and increased the fracture surface roughness. Moreover, addition of SAH/DCP decreased fiber pullout and voids around the fibers by increasing fiber-matrix adhesion. Voids and fiber pull out of the composites would have increased energy dissipation during fracture of composites. It is obvious that voids around fibers increased the path of crack penetration in the transverse direction [8].

## 3. Experimental

### 3.1. Materials

Oil palm empty fruit bunch (EFB) fibers were purchased from Sabutek Sdn Bhd, Malaysia, in the form of short fibers with an average length of 5 mm. The EFB fibers, consisting of 65% cellulose and 19% lignin, were obtained after the removal of oil seeds from fresh fruit bunch used for oil extraction [35]. The fibers were ground and then sieved to the size of 100–200 µm. The sieved fibers were soaked for 3 h and then washed with warm distilled water and acetone, in order to remove impurities, dust and oil from the fibers surface. The washed fibers were then dried in an air oven at 50 °C to a constant weight.

Aliphatic aromatic co-polyester (PBAT), with the trade name of Ecoflex^® ^FBX 7011, was purchased from BASF Plastic Technologies USA. Succinic anhydride (SAH), in white crystalline solid form was supplied by ACRŌS (ORGANICS) Geel (Belgium). Reagent grade chemicals, namely dicumyl peroxide (DCP) and benzoyl peroxide (BPO), used for chemical modification as initiators in producing composites, were provided by Aldrich Chemical Co. Ltd. Chemicals of analytical grade were used throughout the research.

### 3.2. Preparation of the Composite

Preliminary experiments were carried out to prepare new biocomposites and study the effects of fiber loading, on mechanical and thermal properties of EFB fiber/ PBAT composites. The succinic anhydride modified PBAT/EFB biocomposites were prepared using a melt blending technique. Further studies were followed by evaluating the effects of PBAT/EFB biocomposites’ chemical modification using succinic anhydride (SAH) as a coupling agent in the presence and absence of BPO and DCP initiators on the mechanical and thermal properties of PBAT/EFB biocomposites. In addition, SEM and FTIR analyses were carried out to study the effects of chemical treatments on the interfacial characteristics of PBAT/EFB biocomposites.

To study the effect of fiber loading on the thermal and mechanical properties of PBAT biocomposites, the compounding was carried out at different fiber loadings of 10, 20, 30, 40 and 50 (wt %). Thereafter, biocomposites were prepared by compounding PBAT/EFB using 40 (wt %) of EFB fiber based on total weight of composite and different amounts of SAH [0, 1, 2, 3, 4, 5 (wt %)] and peroxides [0, 0.5, 1, 1.5 (wt %)] based on the fiber weight in composites.

The melt blending procedure carried out at 120 °C using a Thermo Haake PolyDrive internal mixer at 30 rpm speed. The composite samples were preheated at 135 °C without applying any pressure for 35 min to allow complete melting. The melted compound was then pressed to mould into sheets at the same temperature. 

### 3.3. FTIR Spectroscopy

A Perkin-Elmer FTIR (model spectrum 100 series) FTIR spectrophotometer was used to determine the functional groups in the samples and to confirm the chemical structure of biocomposites. FTIR spectra tests were run at ambient temperature using a KBr disk method at wave number range of 400 to 4,000 cm^−1^, resolution of 4 cm^−1^.

### 3.4. Biocomposite Characterization

#### 3.4.1. Mechanical Testing of Biocomposites

The produced biocomposite sheets were cut into four standard types of samples for tensile and flexural tests. Tensile tests were carried out according to ASTM Standard Method D638-99 on dumbbell shape specimens with 1 mm thickness, using an Instron Universal Tester (model 4302) at 5 mm/min crosshead speed. The flexural test was performed using the same machine according to ASTM D790-07 on rectangular standard specimens with the dimension of 120 mm × 12.7 mm and 3 mm thickness. Tests were performed at room temperature (about 25 °C) and a minimum of seven samples were tested in each case. 

#### 3.4.2. Thermal Behavior (Thermogravimetric Analysis, TGA)

Neat PBAT and its biocomposites, before and after chemical treatment, were subjected to thermogravimetric analysis using a Perkin–Elmer Thermal Analyzer (model TGA 7). The tests were carried out at a heating rate of 10 °C/min under a nitrogen atmosphere with a flow rate of 20 mL/min. The onset temperature of a 3% (T_3%_) weight loss deviation from the baseline of the thermogravimetric (TG) thermogram was used as the indicator of the composite’s thermal stability and the differential thermogravimetric (DTG) thermograms were recorded to study the weight loss. 

#### 3.4.3. Morphological Features

Tensile fracture surfaces of the composites were examined to evaluate the fiber/matrix adhesion and survey the effect of chemical treatments in different biocomposites, using a scanning electron microscope model LEO 1455VP SEM analyzer. All the surfaces were examined after they were gold coated using a Bal-Tec SDC005 coater sputter.

## 4. Conclusions

Pursuant to the findings presented in this paper, the chemical modification of PBAT/EFB composite using SAH as a coupling agent and DCP to initiate the graft reaction by generating free radicals whilst melt blending, to develop a new biocomposite with high mechanical performances has been successful. The composites’ esterification procedure using 4% SAH at 40% of fiber loading has successfully improved the fiber-matrix interaction, which led to the better incorporation of fiber with the matrix and improved tensile and flexural strength as well as tensile and flexural modulus of the biocomposite in presence of 1% DCP initiator. The mechanical findings corroborated the morphological evidence. The TGA characterization showed thermal stability improvements for PBAT/EFB fiber biocomposite in the presence of DCP initiator, compared to that of PBAT/EFB biocomposite. SEM micrographs demonstrated a better dispersion of EFB fiber into the matrix. SEM provides evidence that the esterification has extensively reduced voids around the fibers, increased interfacial adhesion and presents a uniform surface. 
